# Gender inequality and HIV transmission: a global analysis

**DOI:** 10.7448/IAS.17.1.19035

**Published:** 2014-06-27

**Authors:** Eugene T Richardson, Sean E Collins, Tiffany Kung, James H Jones, Khai Hoan Tram, Victoria L Boggiano, Linda-Gail Bekker, Andrew R Zolopa

**Affiliations:** 1 Division of Infectious Diseases and Geographic Medicine, Stanford University School of Medicine, Stanford, CA, USA; 2 Department of Anthropology, Stanford University, Stanford, CA, USA; 3 Desmond Tutu HIV Centre, University of Cape Town, Cape Town, South Africa

**Keywords:** gender inequality, HIV, AIDS, structural interventions, political ecology

## Abstract

**Introduction:**

The HIV pandemic disproportionately impacts young women. Worldwide, young women aged 15–24 are infected with HIV at rates twice that of young men, and young women alone account for nearly a quarter of all new HIV infections. The incommensurate HIV incidence in young – often poor – women underscores how social and economic inequalities shape the HIV epidemic. Confluent social forces, including political and gender violence, poverty, racism, and sexism impede equal access to therapies and effective care, but most of all constrain the agency of women.

**Methods:**

HIV prevalence data was compiled from the 2010 UNAIDS Global Report. Gender inequality was assessed using the 2011 United Nations Human Development Report Gender Inequality Index (GII). Logistic regression models were created with predominant mode of transmission (heterosexual vs. MSM/IDU) as the dependent variable and GII, Muslim vs. non-Muslim, Democracy Index, male circumcision rate, log gross national income (GNI) per capita at purchasing power parity (PPP), and region as independent variables.

**Results and discussion:**

There is a significant correlation between having a predominantly heterosexual epidemic and high gender inequality across all models. There is not a significant association between whether a country is predominantly Muslim, has a high/low GNI at PPP, has a high/low circumcision rate, and its primary mode of transmission. In addition, there are only three countries that have had a generalized epidemic in the past but no longer have one: Cambodia, Honduras, and Eritrea. GII data are available only for Cambodia and Honduras, and these countries showed a 37 and 34% improvement, respectively, in their Gender Inequality Indices between 1995 and 2011. During the same period, both countries reduced their HIV prevalence below the 1% threshold of a generalized epidemic. This represents limited but compelling evidence that improvements in gender inequality can lead to the abatement of generalized epidemics.

**Conclusions:**

Gender inequality is an important factor in the maintenance – and possibly in the establishment of – generalized HIV epidemics. We should view improvements in gender inequality as part of a broader public health strategy.

## Introduction

The HIV pandemic disproportionately impacts young women. Worldwide, women aged 15–24 are infected with HIV at rates twice that of young men, and young women alone account for nearly a quarter of all new HIV infections. In Sub-Saharan Africa, this rate is even more pronounced: young women account for 31% of new HIV infections [1].

Many reasons have been proposed for the high incidence of HIV in sub-Saharan Africa, including multiple concurrent sex partners [2], income inequality [3], low prevalence of male circumcision [4], and the legacy of colonialism [5]. A UNAIDS/South African Development Community (SADC) think tank – convened to assess prevention in the high prevalence countries of southern Africa – reported that “high levels of multiple and concurrent sexual partnerships by men and women with insufficient consistent, correct condom use, combined with low levels of male circumcision are the key drivers of the epidemic in the sub-region” [6, p. 5]. They concluded by promoting interventions aimed at reducing concurrency and scaling up circumcision. Halperin and Epstein examine these recommendations in regards to the high prevalence in southern and parts of eastern Africa [7]. They highlight that Uganda, Kenya, and Ethiopia were able to achieve significant reductions in HIV incidence through so-called “zero grazing” campaigns (i.e. promotion of monogamy and partner reduction) and suggest the feasibility of implementing these elsewhere. It is worth noting, however, that these countries fare poorly on the Democracy Index [8], and thus such gains may be more realizable in autocratic nations.

The disproportionate HIV incidence in young – often poor – women underscores how social and economic inequalities shape the HIV epidemic. Confluent social forces, including political violence, poverty, racism, and sexism impede equal access to therapies and effective care, *but most of all constrain the agency of women* [9]. Many authors have commented on the role of such forces in driving poor health outcomes for women [10–14], yet there are few examples in the literature of attempts to quantify them (Jewkes and colleagues are a notable exception [15]). This is likely due to the difficulty in quantifying structural interventions [16].

An important question is why HIV epidemics become generalized (i.e., a prevalence greater than 1.0% [17]) in the first place. Circumcision, reduced concurrency, and condom use (the last two of which require agency on the part of women) do not provide a sufficient explanation. In the following analysis, we explore the impact of gender inequality in the establishment and maintenance of heterosexually driven HIV epidemics.

## Methods

**Figure 1:**
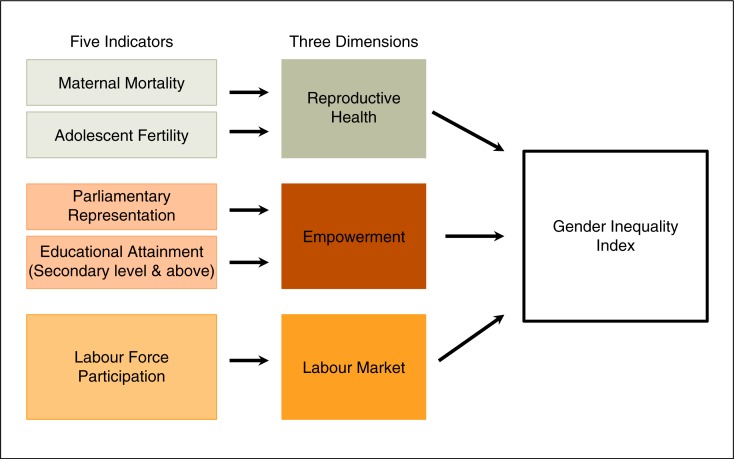
Components of the Gender Inequality Index. (Adapted from the UNDP.)

HIV prevalence data was compiled from the 2010 UNAIDS Global Report [18]. Gender inequality was assessed using the 2011 United Nations Human Development (UNDP) Report Gender Inequality Index (GII) [19]. This indicator is a “composite measure reflecting inequality in achievements between women and men in three dimensions: reproductive health, empowerment, and the labour market” [20,21]. Reproductive health is assessed by the maternal mortality ratio (MMR) and the adolescent fertility ratio (AFR); empowerment, by parliamentary seats held by women and higher educational attainment; and labour market, by women's participation in the workforce (see Figure 1). The index scores nations on a scale from 0 to 1: the higher the index, the more gender inequity there is.

As a preliminary analysis, we regressed log HIV prevalence by country on the GII and calculated a Spearman correlation. Logistic regression models were created to examine the relationship between the predominant mode of HIV transmission (heterosexual vs. MSM/IDU) [22] and factors previously shown or proposed to be predictors of regional HIV incidence: GII, Muslim vs. non-Muslim, Democracy Index, male circumcision rate, and log gross national income (GNI) per capita at purchasing power parity (PPP) [8,19,23–26]. These variables were chosen to cover the main macro-social forces that could lead to increased risk for HIV, viz. gender relations, religion, politics, biology, economics, and geography. Data were analyzed using STATA version 12.1.

## Results and discussion

**Figure 2:**
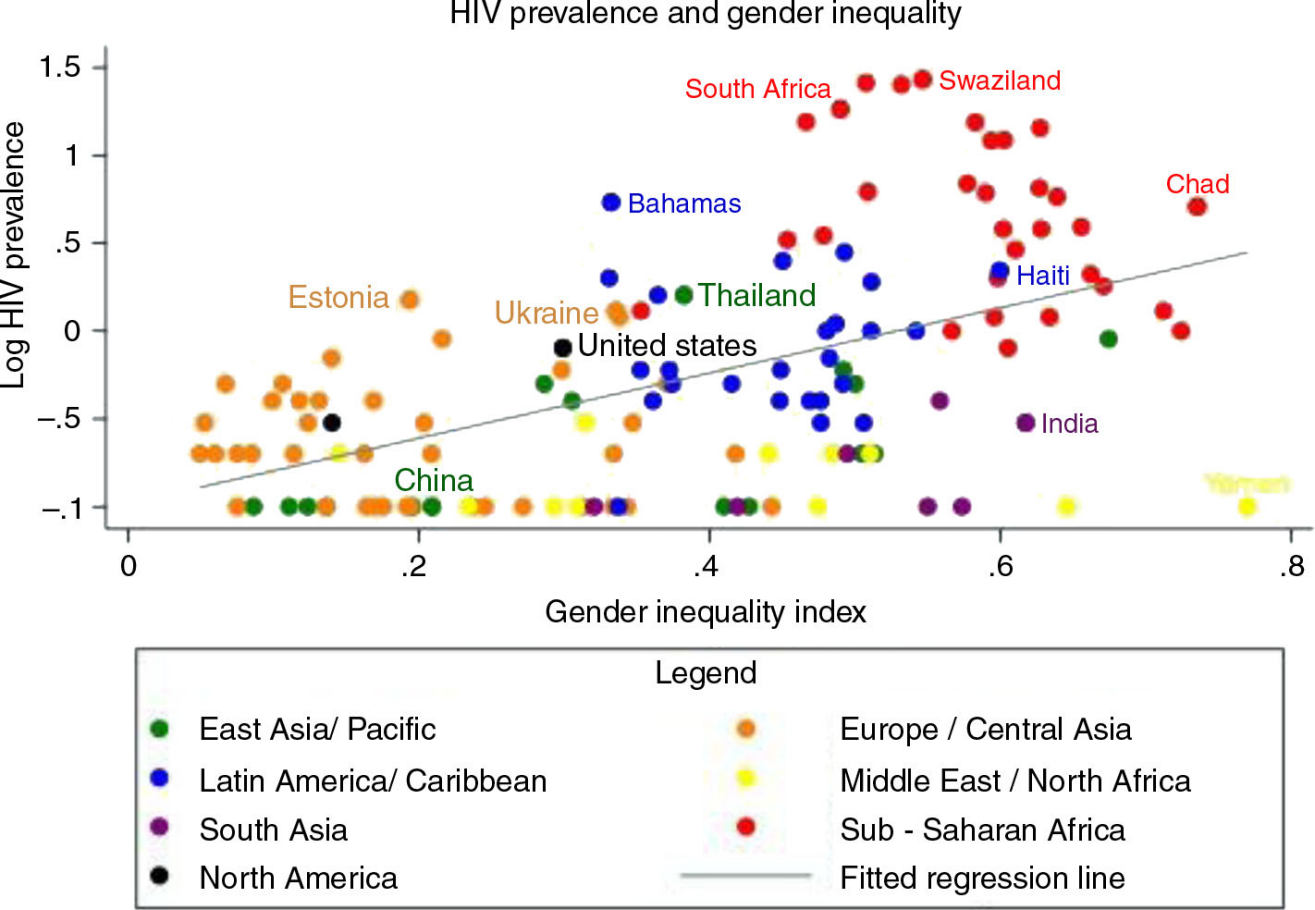
Scatterplot of log HIV prevalence by GII with fitted regression line [18,19]. Sample size=133 countries.

First, we evaluated the log HIV prevalence rates by country using the GII. Figure 2 shows an overall positive correlation between the two variables (Spearman's rho=0.525, *p<*0.001). We found that geographic regions tend to cohere in their prevalence/GII associations. It is notable that there are no countries with a generalized heterosexual epidemic and a GII less than 0.3.

**Figure 3:**
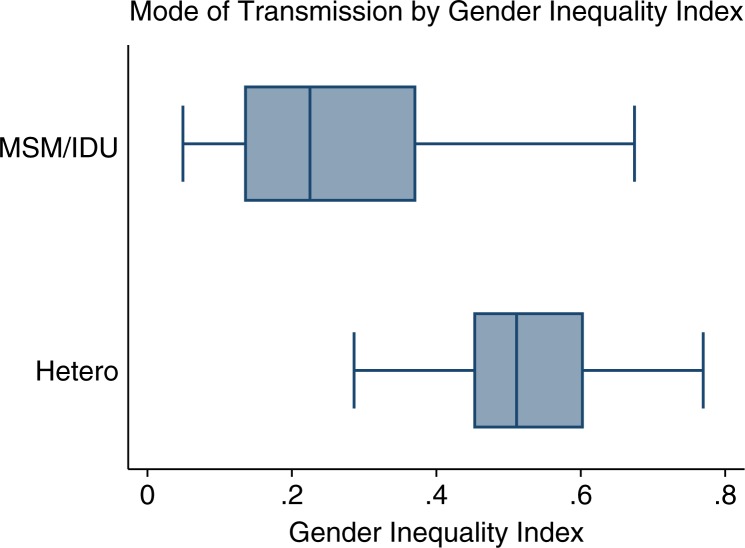
Boxplot of predominant mode of transmission (heterosexual vs. men who have sex with men/injection drug users) by GII [19,23]. Vertical line: median GII; shaded box: interquartile range (IQR); whiskers: span all data points within 1.5 IQR of the nearer quartile. Sample size=133 countries.

Next, we separated countries by predominate type of epidemic – that is, heterosexual vs. non-heterosexual (MSM or IDU). We observed a significant difference (*p<*0.001) in median GII for countries with predominantly heterosexual transmission (0.51) and those where MSM/IDU is the predominant mode (0.23) (Figure 3).

A high GII (i.e., high gender inequality) was a significant predictor of a heterosexual HIV epidemic in our univariate analysis (Table 1, Model 1). We added *a priori* selected known predictors of HIV prevalence sequentially into multivariate logistic regression models of the predominant mode of transmission (Hetero vs. MSM/IDU). In our final multivariate model, GII, Democracy Index, and geographic region were significant predictors of a predominantly heterosexual epidemic (Table 1). After controlling for Muslim vs. non-Muslim, the Democracy Index, male circumcision rate, log GNI per capita at PPP, and region, a country is 2.34 times more likely to have a heterosexually driven epidemic for every 0.1 increase in GII. Since the GII ranges from 0.07 to 0.77, countries with severe gender inequality are over 15 times more likely to have a heterosexually driven epidemic compared to countries with near gender parity.

**Table 1:** Logistic regression models with predominant mode of transmission (heterosexual vs. MSM/IDU) as the dependent variable and the GII, Muslim vs. non-Muslim, Democracy Index, male circumcision rate, log GNI per capita at PPP, and region as independent variables [8,19,23–26]

	Model 1	Model 2	Model 3	Model 4	Model 5	Model 6
GII	3.40 (2.30–5.04) [6.12***]	3.68 (2.34–5.81) [5.63***]	3.54 (2.10–5.96) [4.74***]	3.32 (1.97–5.61) [4.50***]	3.14 (1.62–6.11) [3.37**]	2.34 (1.15–4.74) [2.35*]
Muslim vs. non-Muslim		5.87 (1.58–21.9) [2.64**]	2.24 (0.49–10.3) [1.04]	0.75 (0.06–8.96) [−0.22]	0.64 (0.05–8.35) [−0.34]	1.63 (0.10–27.6) [0.34]
Democracy Index			0.65 (0.46–0.93) [−2.34*]	0.65 (0.46–0.94) [−2.32*]	0.63 (0.40–0.97) [−2.11*]	0.62 (0.39–0.97) [−2.09*]
Male circumcision				1.01 (0.99–1.04) [1.12]	1.02 (0.99–1.04) [1.16]	1.00 (0.97–1.03) [0.19]
Log GNI per capita at PPP					0.99 (0.15–6.52) [−0.01]	0.62 (0.07–5.32) [−0.44]
Region						1.68 (1.10–2.55) [2.42*]
Sample size	133	133	129	129	124	124
AIC	110	104	92	93	91	86
Pseudo R-square	0.43	0.47	0.53	0.54	0.54	0.58

Unbracketed numbers represent odds ratios. Numbers in parenthesis are 95% confidence intervals. Numbers in brackets are z-scores.

* *p<*0.05

** *p<*0.01

*** *p<*0.001.

Being a country in the African region is also strongly associated with having a predominantly heterosexual epidemic. This may be a marker for concurrent relationships [2,7, 27–29], or for geographic proximity to the emergence of the virus. In contrast, Central Asia and Eastern Europe are predominantly IDU driven while epidemics in Latin America, North America, Western Europe, and Australia tend to be driven by men who have sex with men.

More democratic countries are less likely to have a heterosexually driven epidemic. This may reflect an association between better gender equality and a higher level of democracy by country, which is not fully captured by the GII. Our final model did not show a significant association between a country's predominate mode of transmission whether it is predominantly Muslim, has a high/low GNI at PPP, or has a high/low circumcision rate.

## Conclusions

This analysis demonstrates that predominantly heterosexually driven epidemics are associated with higher Gender Inequality Indices (i.e., worse gender inequality) in comparison to countries where MSM or IDU are the primary mode of transmission. This association holds up even after including other social and economic factors that have been shown to impact a country's HIV epidemic. The countries of the Middle East and North Africa are a notable exception: they uniformly show high Gender Inequality Indices coupled with a low HIV prevalence. Others have related this to the influence of circumcision as well as a protective effect of Islamic social organization in relation to a low rate of partner concurrency [30]. This is difficult to empirically verify, however, since reliable global data on concurrency are lacking.

Further evidence that supports the role that gender inequality plays in generalized HIV epidemics comes from looking at temporal trends in countries that have had a generalized epidemic in the past but no longer have one. Using UNAIDS high estimates for prevalence, there are only three countries that fit this characterization: Cambodia, Honduras, and Eritrea [18]. GII data are available only for Cambodia and Honduras, and these countries showed a 37 and 34% improvement, respectively, in their Gender Inequality Indices between 1995 and 2011 [19]. During the same period, both countries reduced their HIV prevalence below the 1% threshold of a generalized epidemic. *This represents limited but compelling evidence that improvements in gender inequality can lead to the abatement of generalized epidemics*. It also suggests that we see improvements in gender inequality as a part of a broader public health strategy [31]. That the prevalence of forced first sex among adolescent girls younger than 15 years ranges between 11 and 48% globally [32], however, is a stark reminder that we have a long way to go to achieving gender equality.

Possible mechanisms for the maintenance of generalized heterosexual epidemics by gender inequality include transactional sex, rape and domestic violence, intergenerational sex, inability to negotiate condom use, decreased access to care, and limited opportunities for education leading to a misapprehension of risk.

Examples of structural interventions in gender inequality include abolishing school fees for girls, *real* enforcement of domestic violence and rape laws, affirmative action for increased participation in the labour market, government grants [33], and banning child marriage. Such interventions could be critically important for countries like India, where high gender inequality may the harbinger of a future generalized heterosexual epidemic. Indeed, our findings suggest India's current campaign against domestic violence and rape may have added public health benefits in terms of HIV prevention.

In short, the GII was invented to “help governments and others understand the ramifications of gaps between women and men” [21]. As an example, Singh and colleagues have shown a positive association between the GII and both cervical cancer incidence and mortality. They conclude by stating, “reductions in cervical cancer rates are achievable by reducing inequalities in socio-economic conditions … and women's social status” [34, p. 17]. Similarly, our analysis suggests that gender inequality is an important factor in the maintenance – and possibly in the establishment – of generalized HIV epidemics and thus provides an important reminder that we may not be able to treat ourselves out of the pandemic with medications alone.

## Limitations

There is a potential ecological fallacy in using aggregate data like the GII to make inferences about the individual acts whereby HIV is transmitted; however, approaching the analysis within a theoretical framework can isolate meaningful structural forces [35–36]. This is in opposition to the shotgun/every-variable approach [37], where it becomes difficult to disentangle meaningful mechanisms of transmission.

The GII is limited to data available to the UNDP. Thus, the political empowerment component only indexes parliamentary participation and fails to characterize involvement elsewhere in community and public life. The labour market component does not capture the time women spend in unpaid or informal labour. Lastly, gender-based violence and participation in community decision-making are not adequately indexed [38].

In addition, there may be some confounders that were not adequately accounted for in our analysis. For example, the fact that the overall pandemic had its origins on the African continent may have allowed continents that were affected later (e.g. Asia) time to develop a more adequate prevention response. However, that South Africa had a relatively late epidemic (comparable to Thailand) – yet still has one of the highest HIV prevalences in the world – argues somewhat against this factor having a major impact.

Finally, the designation of a predominant mode of transmission is not so clear-cut in some countries and can change over time. For example, the epidemic in China was previously driven by IDU transmission but is now predominantly heterosexual [39].

Despite these potential limitations we feel that our analysis demonstrates compelling evidence that gender inequality is an important factor driving heterosexual and, therefore, more generalized HIV epidemics. Public health policy focused on reducing HIV prevalence in generalized epidemics should consider structural interventions that address gender inequality.

## References

[1] UNAIDS (2012). Fact sheet: adolescents, young people and HIV.

[2] Kenyon C, Buyze J, Colebunders R (2013). HIV prevalence by race co-varies closely with concurrency and number of sex partners in South Africa. PLoS One.

[3] Holmqvist G (2009). HIV and income inequality: if there is a link, what does it tell us?. International Policy Centre for Inclusive Growth (UNDP) working paper.

[4] Mehta SD, Moses S, Agot K, Odoyo-June E, Li H, Maclean I (2013). The long term efficacy of medical male circumcision against HIV acquisition. AIDS.

[5] Barnett T, Whiteside A (2002). AIDS in the twenty-first century: disease and globalization.

[6] Southern African Development Community (SADC) Meeting Report (2006). Experts Think Tank Meeting on HIV Prevention in High-Prevalence Countries in Southern Africa; , 2006 in Maseru, Lesotho.

[7] Halperin DT, Epstein H (2007). Why is HIV prevalence so severe in Southern Africa? The role of multiple concurrent partnerships and lack of male circumcision: implications for AIDS prevention. S Afr J HIV Med.

[8] Economist Intelligence Unit (2006). Democracy Index [Internet]. http://www.economist.com/media/pdf/DEMOCRACY_TABLE_2007_v3.pdf.

[9] Farmer P (2001). Infections and inequalities: the modern plagues.

[10] Farmer PE, Nizeye B, Stulac S, Keshavjee S (2006). Structural violence and clinical medicine. PLoS Med.

[11] Mukherjee JS, Barry DJ, Satti H, Raymonville M, Marsh S, Smith-fawzi MK (2011). Structural violence : a barrier to achieving the millenium development goals for women. J Womens Health.

[12] Abdool Karim Q, Sibeko S, Baxter C (2010). Preventing HIV infection in women: a global health imperative. Clin Infect Dis.

[13] Nattrass N (2009). Poverty, sex and HIV. AIDS Behav.

[14] Epstein H, Kim J (2007). AIDS and the power of women. New York Rev Books.

[15] Jewkes RK, Dunkle K, Nduna M, Shai N (2010). Intimate partner violence, relationship power inequity, and incidence of HIV infection in young women in South Africa: a cohort study. Lancet.

[16] Bertozzi SM, Gutiérrez J-P (2013). Poverty, cash transfers, and risk behaviours. Lancet Glob Health.

[17] World Health Organization (2002). Second generation surveillance for HIV/AIDS [Internet]. http://www.who.int/hiv/topics/surveillance/2ndgen/en/.

[18] (2010). UNAIDS report on the global AIDS epidemic [Internet]. http://www.unaids.org/globalreport/global_report.htm.

[19] United Nations Development Programme (UNDP), Gender Inequality Index Trend 1995–2011 (2011). http://hdr.undp.org/en/media/Gender_Inequality_Index_Trend_1995–2011.xls.

[20] United Nations Development Programme (UNDP) human development indicators [Internet]. http://hdrstats.undp.org/en/indicators/68606.html.

[21] United Nations Development Programme (UNDP) Components of the Gender Inequality Index [Internet]. http://hdr.undp.org/en/statistics/gii/.

[22] UNAIDS (2009). AIDS epidemic update.

[23] UNAIDS (2002). Report on the global HIV/AIDS epidemic.

[24] Pew Research Center Muslim population by country [Internet]. http://features.pewforum.org/muslim-population/.

[25] Waskett J (2012). Global circumcision rates [Internet]. http://www.circs.org/index.php/Reviews/Rates/Global.

[26] World Bank, International Comparison Program database Gross National Income per capita at purchasing power parity [Internet]. http://data.worldbank.org/indicator/NY.GNP.PCAP.PP.CD.

[27] Goodreau SM (2011). A decade of modelling research yields considerable evidence for the importance of concurrency: a response to Sawers and Stillwaggon. J Int AIDS Soc.

[28] Eaton JW, Hallett TB, Garnett GP (2011). Concurrent sexual partnerships and primary HIV infection: a critical interaction. AIDS Behav.

[29] Mah TL, Halperin DT (2010). Concurrent Sexual Partnerships and the HIV Epidemics in Africa: Evidence to Move Forward. AIDS Behav.

[30] Gray PB (2004). HIV and Islam: is HIV prevalence lower among Muslims?. Soc Sci Med.

[31] Richardson ET (2014). Research on oral pre-exposure prophylaxis in sub-Saharan Africa is an example of biomedical tunnel vision. AIDS.

[32] World Health Organization (2005). WHO multi-country study on women's health and domestic violence against women: summary report of initial results on prevalence, health outcomes and women's responses.

[33] Cluver L, Boyes M, Orkin M, Pantelic M, Molwena T, Sherr L (2013). Child-focused state cash transfers and adolescent risk of HIV infection in South Africa: a propensity-score-matched case-control study. Lancet Global Health.

[34] Singh GK, Azuine RE, Siahpush M (2012). Global inequalities in cervical cancer incidence and mortality are linked to deprivation, low socioeconomic status, and human development. Int J MCH AIDS.

[35] Kelly M (2010). The role of theory in qualitative health research. Fam Pract.

[36] Bourdieu P (1977). Outline of a Theory of Practice.

[37] Drain PK, Smith JS, Hughes JP, Halperin DT, Holmes KK (2004). Correlates of national HIV seroprevalence. J Acquir Immune Defic Syndr.

[38] UNDP (2013). FAQ – Gender Inequality Index (GII) [Internet]. Human development reports.

[39] Wang L, Wang N, Wang L, Li D, Jia M, Gao X (2009). The 2007 estimates for people at risk for and living with HIV in China: progress and challenges. J Acquir Immune Defic Syndr.

